# Origin and diversity of the wild cottons (*Gossypium hirsutum*) of Mound Key, Florida

**DOI:** 10.1038/s41598-024-64887-8

**Published:** 2024-06-18

**Authors:** Weixuan Ning, Karen M. Rogers, Chuan-Yu Hsu, Zenaida V. Magbanua, Olga Pechanova, Mark A. Arick, Ehsan Kayal, Guanjing Hu, Daniel G. Peterson, Joshua A. Udall, Corrinne E. Grover, Jonathan F. Wendel

**Affiliations:** 1 Ecology, Evolution, and Organismal Biology Department, Iowa State University, Ames, IA 50011 USA; 2 Division of Recreation and Parks, Florida Department of Environmental Protection, District 4 Administration, 1843 S. Tamiami Trail, Osprey, FL 34229 USA; 3 Institute for Genomics, Biocomputing & Biotechnology, Mississippi State University, Starkville, MS 39762 USA; 4 National Key Laboratory of Cotton Bio-Breeding and Integrated Utilization, Institute of Cotton Research, Chinese Academy of Agricultural Sciences, Anyang, 455000 China; 5 Shenzhen Branch, Guangdong Laboratory of Lingnan Modern Agriculture, Key Laboratory of Synthetic Biology, Ministry of Agriculture and Rural Affairs, Agricultural Genomics Institute at Shenzhen, Chinese Academy of Agricultural Sciences, Shenzhen, 518120 China; 6 Crop Germplasm Research Unit, USDA/Agricultural Research Service, 2881 F&B Road, College Station, TX 77845 USA

**Keywords:** Domestication, Genetic diversity, *Gossypium hirsutum*, Polyploid, Upland cotton, Wild relatives, Agricultural genetics, Evolutionary genetics

## Abstract

Elucidating genetic diversity within wild forms of modern crops is essential for understanding domestication and the possibilities of wild germplasm utilization. *Gossypium hirsutum* is a predominant source of natural plant fibers and the most widely cultivated cotton species. Wild forms of *G. hirsutum* are challenging to distinguish from feral derivatives, and truly wild populations are uncommon. Here we characterize a population from Mound Key Archaeological State Park, Florida using genome-wide SNPs extracted from 25 individuals over three sites. Our results reveal that this population is genetically dissimilar from other known wild, landrace, and domesticated cottons, and likely represents a pocket of previously unrecognized wild genetic diversity. The unexpected level of divergence between the Mound Key population and other wild cotton populations suggests that the species may harbor other remnant and genetically distinct populations that are geographically scattered in suitable habitats throughout the Caribbean. Our work thus has broader conservation genetic implications and suggests that further exploration of natural diversity in this species is warranted.

## Introduction

The plant domestication process entails strong artificial selection based on desirable phenotypic traits, resulting in a genetically narrowed gene pool and often spatial isolation and phenological divergence from their wild relatives^1,2^. Wild relatives of cultivated crops thus can contain diversity that has not been captured in modern breeding populations^3^, with this diversity being found in often widely scattered or relictual populations that were not involved in the source pools of the cultivated forms.

This scenario is particularly applicable to the dominant cultivated cotton species, *Gossypium hirsutum* (genome designation AD_1_), which is an allopolyploid derived from hybridization and whole genome duplication (WGD) of two diverged diploids (genomes A and D) around 1 to 2 mya (million years ago)^4,5^. After the initial WGD event, the nascent polyploid lineage underwent diversification and speciation, generating seven recognized species, including two that were ultimately domesticated (i.e., *G. hirsutum* and *G. barbadense*)^4,6^. Modern domesticated *G. hirsutum*, comprising over 90% of the cotton grown worldwide, is planted as an annualized row crop for its abundant fine “fiber”, which botanically consists of elongated single-celled seed trichomes that develop from ovular epidermal cells^7^. Initial domestication is thought to have occurred in the northern Yucatan peninsula^8,9^, with subsequent geographic expansion throughout the American tropics in pre-colonial times. Human-mediated domestication has led to multiple changes in plant habit, phenology (e.g., perennial vs annual), and growth form (e.g., shrub to large tree vs large herbaceous row-crop)^7,10,11^.

**Figure 1:**
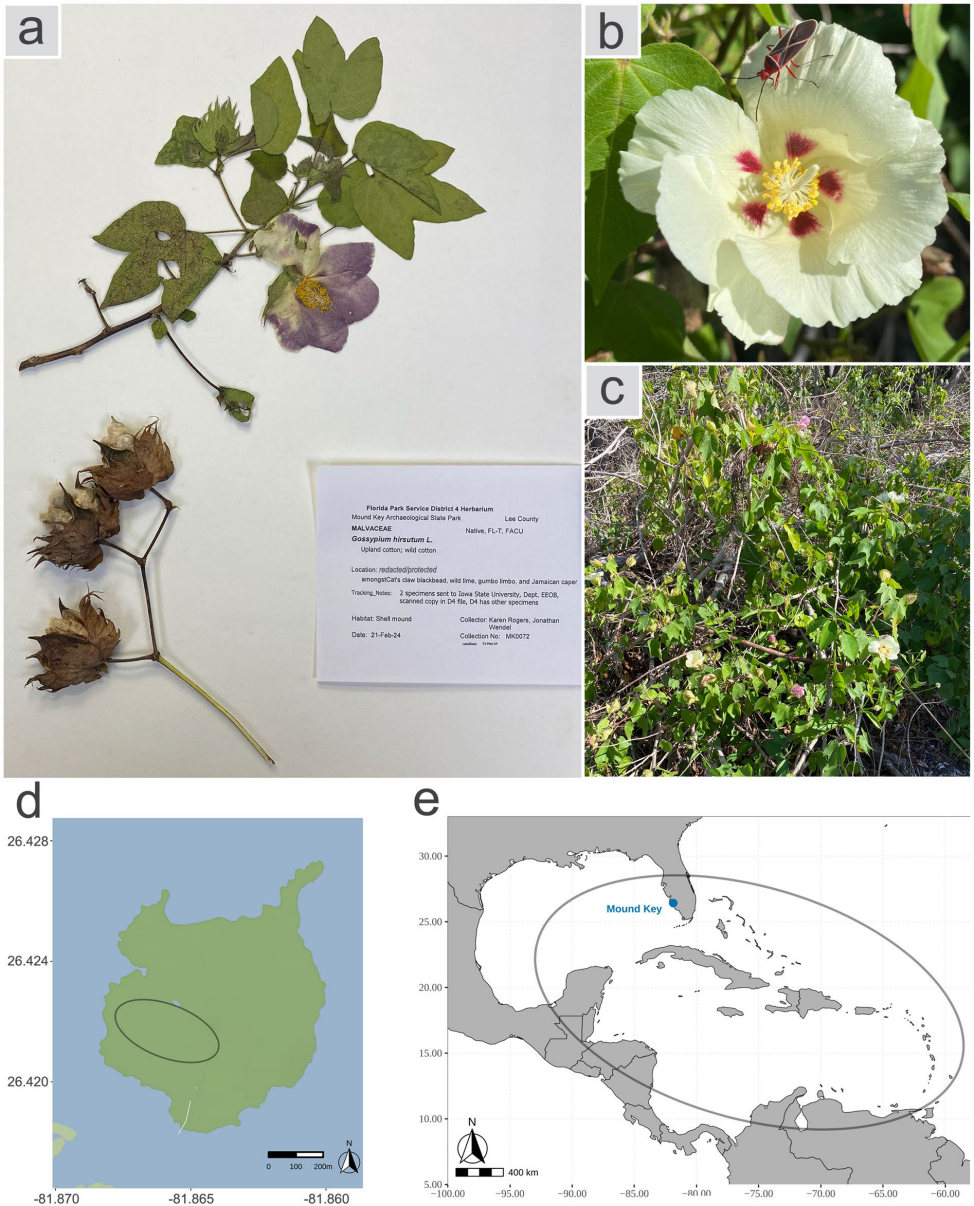
Illustrative morphological features and distribution of Mound Key *Gossypium hirsutum*. (**a**) An herbarium sheet for Mound Key cotton. (**b**,**c**) Field-taken photos showing whole plant and flower. (**d**) Sampling of *Gossypium hirsutum* at three sites on Mound Key, Florida, US. Three different sites within the black circle were sampled, within several hundred meters of each other. Exact locations are obscured to protect the threatened status of the species. In total, 25 individuals were sampled, including 17 from Site 1, 7 from Site 2, and 1 from Site 3. The map was generated using Stadia Maps (https://docs.stadiamaps.com/) and ggmap (v.4.0.0)^53^ package in R. (**e**) Distribution of wild cotton (within black circle) in the Caribbean. The map was generated using rnaturalearth (v.1.0.1) (https://docs.ropensci.org/rnaturalearth/) package in R.

As a wild species, *G. hirsutum* is relatively rare, because of both intentional (human activities) and unintentional (habitat loss) eradication. Presently, the species is restricted to the drier coastal areas of the northern Yucatan Peninsula, SW Florida, SW Puerto Rico, and a number of additional islands throughout the Caribbean (Fig. 1e)^12,13^. Although the aggregate geographical distribution is quite broad, ecological niche modeling shows that wild populations occupy narrow and scattered habitats within this extensive range^12,14^.

It is often challenging to determine whether a natural cotton population is truly “wild”. Feral derivatives are common throughout and beyond the native range of the species, having become reestablished in natural vegetation over the several millennia of cotton domestication and diffusion across cultures as it spread to encompass much of the American tropics and subtropics. Some of these feral derivatives exhibit morphological characteristics that broadly overlap with those of wild forms^6,12,13^, rendering the diagnosis of status (wild vs feral) especially challenging.

Some clarity into the wild vs feral cotton problem has emerged from species-wide analyses of molecular markers^8,9^, the most recent being the relatively comprehensive genomic survey by Yuan et al.^15^ that provides a framework for placing “unknown samples” with respect to their domestication status (e.g., truly wild vs feral) and genetic relationships. Using single nucleotide polymorphisms (SNPs) data extracted from whole genome resequencing, Yuan et al.^15^ revealed four genetically distinct groups: one group consisting of wild forms; two clusters of primitively domesticated and feral populations, termed landrace1 (abbreviation LR1; most of islands around Caribbean Sea) and landrace2 (abbreviation LR2; Central America); and modern crop cultivars (globally). Given the relative paucity of arguably wild forms in germplasm collections, relatively few were included in Yuan et al.^15^, which likely insufficiently capture the scope of genetic diversity within wild *G. hirsutum*. Increasing the sampling of natural populations, therefore, is crucial for a better understanding of *G. hirsutum* as a wild species today.

Mound Key (abbreviation MK), located on the SW coast of Florida (USA), is an isolated small island (51 hectare) that harbors a population of *G. hirsutum*, fully integrated into the native vegetation and a morphology characteristic of wild plants (shrubby to tree-like habit, small flowers and capsules, sparse and shorter brownish seed hairs) (Fig. 1). This suggests that the population in Mound Key may truly be wild *G. hirsutum*. On the other hand, Mound Key was the historical political capital of the Calusa kingdom over 2000 years ago, established from discarded shells, bone, and pottery, rising up to 9 m above the surrounding Estero Bay^16^. The Calusa might have cultivated cotton for their net-fishing culture, which started about 6000 years ago around Useppa Island in southern Florida^17^. In addition, in the late 19th/early 20th century, settlers from the Koreshan Unity maintained agricultural lands on Mound Key. Thus, the Mound Key cotton may be feral instead of wild.

In this paper, we performed whole genome resequencing of 25 individuals sampled from three sites on Mound Key (Fig. 1d) to address several questions: (1) Can we determine if the Mound Key population is truly wild, as opposed to being a feral derivative tracing to Calusa culture, by incorporating the new genome sequence data into the phylogenomic framework established earlier^15^? (2) What are the genetic relationships of the Mound Key population to the broader *G. hirsutum* gene pool? (3) How much of the broader genetic diversity is represented by the Mound Key population? (4) How much genetic diversity exists in this single population, and how is it distributed within and among individuals? Our results provide new insights into the nature of wild cotton, and foreshadow an increasing awareness of the true scope of genetic diversity among wild populations.

## Results

### Whole genome sequencing of Mound Key cotton

For the 25 *G. hirsutum* samples collected from three sites on Mound Key, we performed whole genome sequencing with an average depth of ~ 23.6x (range 18.6x–30.2x) across the reference genome (Table S1). In the joint genotyping analysis that included these MK samples and 40 representatives selected from the four previously designated groups of *G. hirsutum* (i.e., wild, LR1, LR2, cultivar^15^), we identified approximately 1.9 billion (B) invariable sites (> 2 B sites total), 7.6 million (M) SNP sites, and 4.4 M small insertion and deletion (indels) (Table 1). The total number of sites and invariable sites was 1.6 times greater in the At subgenome compared to the Dt subgenome, with more than twofold differences in SNPs sites and indels.

**Table 1:** Total number of unfiltered sites from two subgenomes of *Gossypium hirsutum* (At and Dt) across all 65 sampled individuals, as well as the number of filtered invariable, SNP, and indel sites.

	Total	Invariable	SNP	Indel
At subgenome	1,436,043,920	1,163,364,035	5,091,924	2,529,137
Dt subgenome	848,680,376	717,923,537	2,488,966	1,910,087
Sum	2,284,724,296	1,881,287,572	7,580,890	4,439,224

### Population genetic relationships of *G. hirsutum*

**Figure 2:**
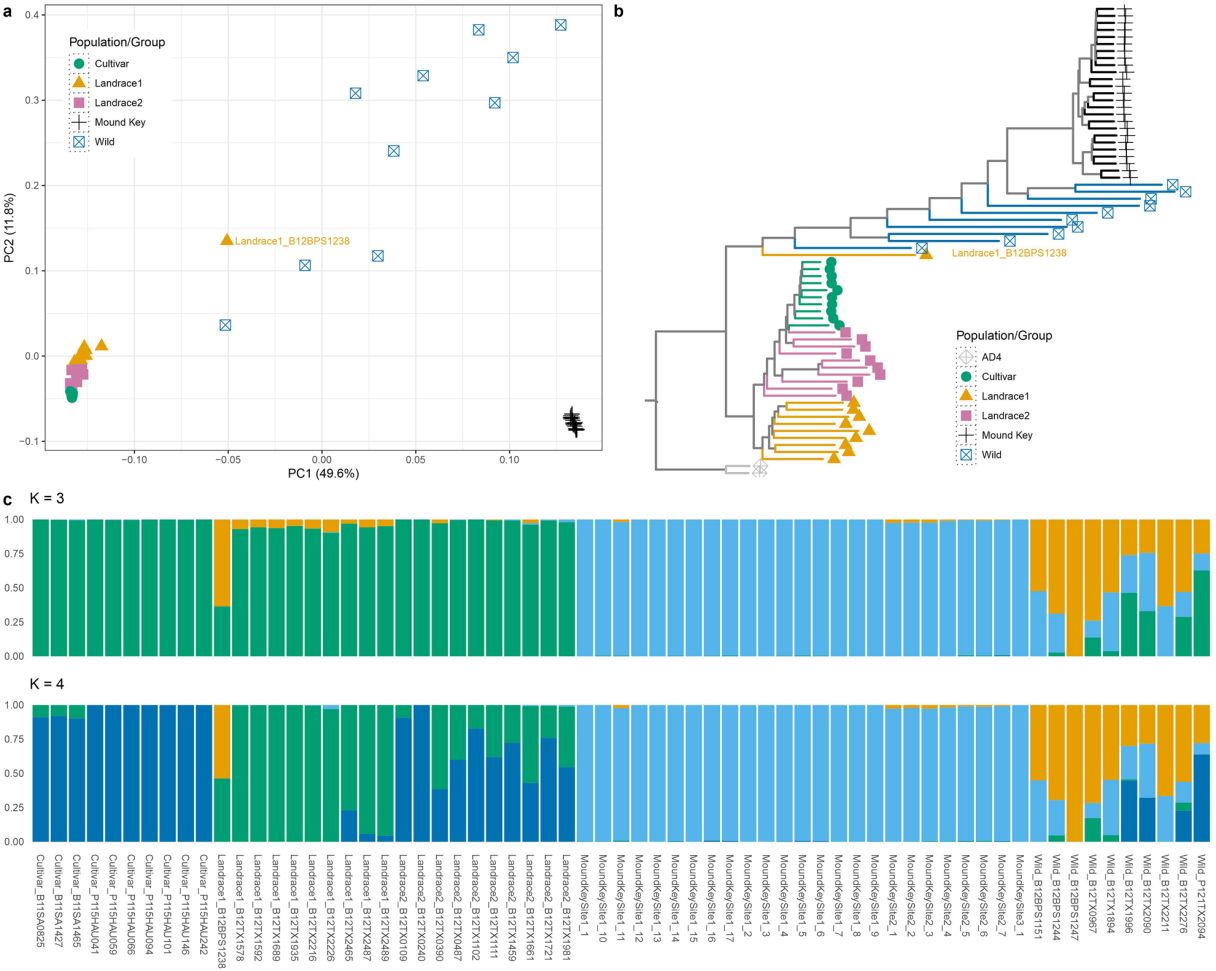
Population genetic structure analysis of Mound Key and four a priori designated groups of *Gossypium hirsutum*, including cultivars, landrace 1 & 2, and wild cottons, using 11, 539 unlinked genic SNPs. Each group is represented by a different color and shape in (**a**) PCA and (**b**) rooted neighbor-joining phylogenetic tree. (**c**) LEA genetic structure for three or four ancestral populations (K = 3 and K = 4). Each bar is labeled by an individual sample name (i.e., Accession/Group_SampleID) and filled with different lengths of colors corresponding to proportions of ancestral population signals.

To determine the status of the MK population as being either feral or wild, we first examined these samples in the broader context with the *G. hirsutum* germplasm pool reported in Yuan et al.^15^. Principal component analysis (PCA) using 11,320 filtered unlinked and genic SNPs (out of total 7,580,890 SNPs) recovered two tightly knit clusters alongside a spread of dispersed samples. PC1 explained nearly half (49.6%) of total among sample variation and generally divided the samples into three groups: a domesticated cluster comprising cultivars and both landraces positioned on the lower left; a Mound Key cluster on the lower right; and wild samples widely dispersed between the two (Fig. 2a). The PC2 explained 11.8% of the variation and primarily separated the previously sequenced wild samples (Fig. 2a). A single sample from LR1 overlapped with the wild samples on the first axis, potentially revealing an early cultivar or introgression in that accession (discussed later). Neighbor-joining analysis using 10,322 LD pruned genic SNPs across 67 samples (65 *G. hirsutum* and two *G. mustelinum* as the outgroup) reiterated the PCA results, with MK samples forming a monophyletic clade branching close to the majority of the wild cottons, which were paraphyletic (Fig. 2b). The cultivars formed a monophyletic clade with LR2, which was sister to LR1.

Genetic structure analysis via LEA suggests either three or four ancestral population clusters among the sampled 65 *G. hirsutum* individuals (Fig. 2c; Fig. S2) that differ in their resolution among the domesticated cottons. When K = 3, the results mirror those from PCA and the neighbor-joining tree, where all three groups with domesticated histories originate from the same ancestral population, excluding one landrace sample (Landrace1_B12BPS1238), MK samples are derived from a different population, and wild samples exhibit mixed ancestry. When the number of projected ancestral populations is increased (K = 4), additional structural differences emerge between the LR1, LR2, and cultivar samples while the Mound Key samples remain distinct.

### Diversity and divergence of the Mound Key cotton

We evaluated the diversity within Mound Key cotton and its distance from the other predesignated groups. As a potentially highly inbred population (due to small size and geographical isolation on a relatively small island), we calculated the extent of heterozygosity and inbreeding level using all filtered SNPs (Table S2), and compared MK to the previously designated groups. As expected, the wild samples exhibited the greatest proportion of heterozygosity He (0.14; Table 2) and the lowest inbreeding coefficient F_IS_ (0.56), whereas the cultivars exhibited the lowest He (0.04) and the highest F_IS_ (0.88). As the likely source of the cultivars, it is perhaps unsurprising that LR2 exhibited heterozygosity (He = 0.07) and inbreeding (F_IS_ = 0.78) closer to the cultivated cottons. Notably, heterozygosity and inbreeding for MK was most similar to both wild cottons and LR1 (Table 2), potentially indicating that MK has experienced a similar level of inbreeding as experienced by LR1 and wild cottons.

**Table 2:** Diversity and inbreeding in populations of *Gossypium hirsutum*.

	Avg_He ± SD	FIS ± SD	π
Cultivar	0.04 ± 0.02	0.88 ± 0.05	2.60 × 10^–4^
Landrace1 (LR1)	0.13 ± 0.09	0.61 ± 0.29	7.60 × 10^–4^
Landrace2 (LR2)	0.07 ± 0.03	0.78 ± 0.1	7.70 × 10^–4^
Mound Key (MK)	0.13 ± 0.01	0.6 ± 0.04	4.30 × 10^–4^
Wild	0.14 ± 0.06	0.56 ± 0.19	9.90 × 10^–4^

Average observed heterozygosity sites (Avg_He), nucleotide diversity (π), and Wright's inbreeding coefficient (F_IS_) among all filtered 7,580,890 SNPs for each group, with standard deviation (SD). Cultivar, LR1, LR2 and wild groupings are derived from Yuan^15^.

**Figure 3:**
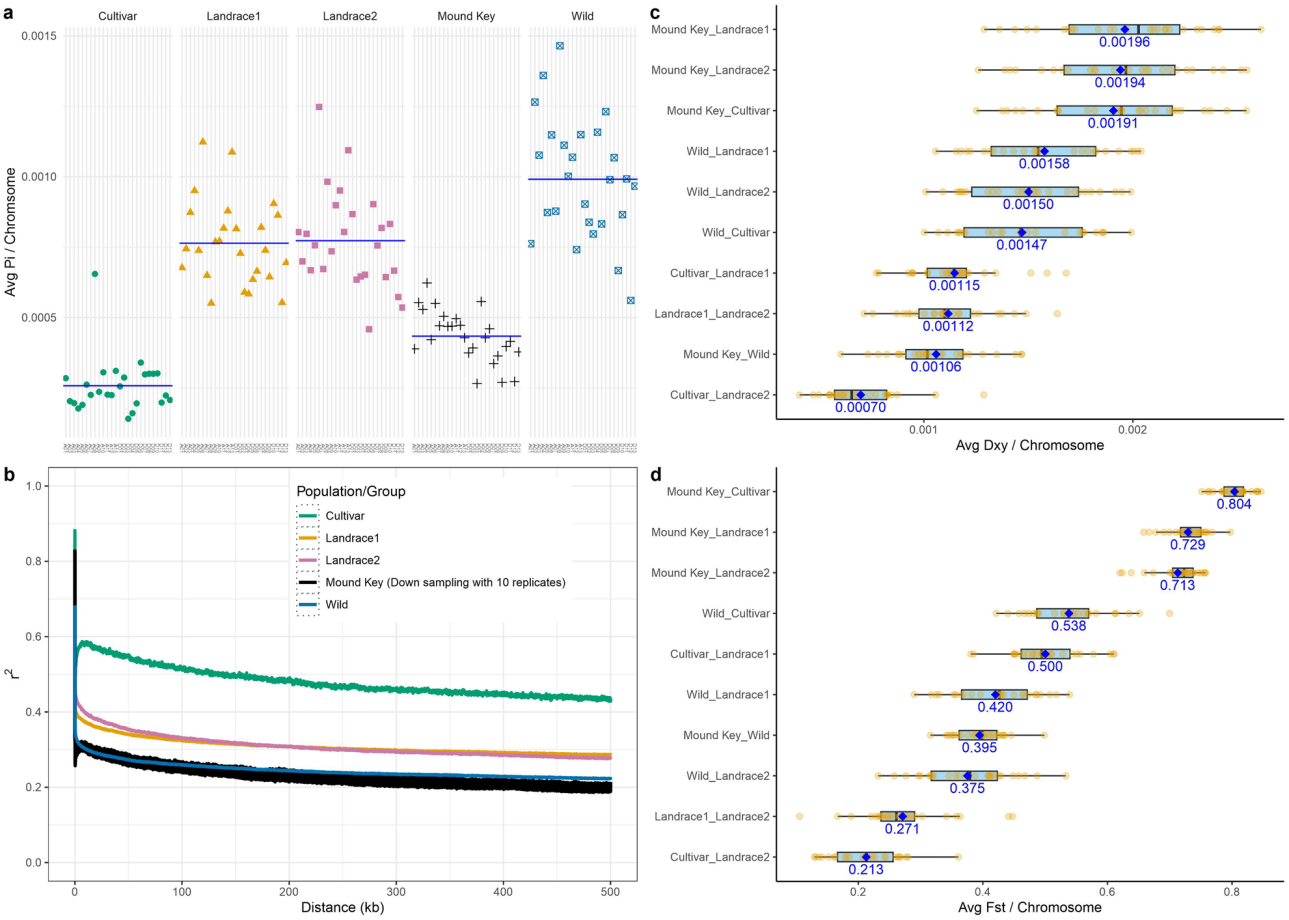
Measures of diversity and divergence from Mound Key population and four designated genetic groups (i.e., landrace1, landrace2, and cultivar). (**a**) Averaged nucleotide diversity (π) per chromosome. Populations are distinguished by shape and color. Chromosomes are ordered along the x-axis the At to Dt chromosomes. Mean π across all chromosomes is indicated by a blue line. (**b**) The decay rate of linkage disequilibrium (*r*^*2*^; y-axis) for pairs of SNPs as physical distance (bp) increases (x-axis). Each color represents one group, with Mound Key represented by ten black lines for each down-sampled set. (**c**) Per chromosome pairwise averaged dxy and (**d**) Fst comparisons between the four genetic groups and Mound Key (yellow dots). The overall average dxy and Fst (x-axis) between each pair-wise comparison group (y-axis) is shown by a blue diamond shape within the boxplot and actual values underneath each boxplot.

We also measured overall genetic diversity (Table2; Fig. 3a) for the MK population and four predesignated groups. As expected, nucleotide diversity was greatest in the wild cottons (π = 9.9 × 10^–4^). The MK samples exhibited only twofold lower diversity than the wild samples (π = 4.3 × 10^–4^), a level of diversity that is intermediate between cultivar samples (π = 2.6 × 10^–4^) and the two landrace groups (π ~ 7.6 × 10^–4^ for each), but we regard this diversity as notable given the spatial isolation from the other wild cottons and the small size of the MK population.

In an effort to quantify the genetic divergence of MK and four groups from each other, we calculated all pairwise nucleotide differences (dxy) (Fig. 3c). Notably, MK exhibits fewer differences with the wild samples (dxy ~ 1 × 10^−3^) versus either of the two landrace groups (*c.* 1.95 × 10^−3^). Moreover, dxy between MK and any of the domesticated groups was even greater than between the wild group versus any of the domesticated groups (Fig. 3c). Among the three domesticated groups, both cultivar and LR2 showed sequence divergence that were more similar to each other (dxy = *c.* 0.7 × 10^−3^) than either one was to LR1 (*c.* 1.13 × 10^−3^). These data support the distinctiveness of MK, relative to other groups, and MK’s close relationship to the wild group.

Overall, Fst among the four groups ranged from 0.21 (LR2 versus domesticated) to 0.80 (MK versus cultivar; Fig. 3d), the higher end of the range reflecting comparisons between MK versus any of the three groups with a history of domestication (Fst > 0.7). Although the pairwise Fst between MK and the wild cottons was the lowest for all MK Fst comparisons (0.395; Fig. 3d), it was similar in value to the Fst between wild and either landrace group (*c.* 0.40), perhaps reflecting the isolated nature of this population.

We evaluated the level of linkage disequilibrium (LD) decay for MK and the other groups to provide additional perspective on their demographic history. As expected, the cultivars maintain a high level of LD (*r*^*2*^ > 0.4) even across physical distances greater than 500 kbp (Fig. 3b). By contrast, LD for the two landraces showed a decay rate that was slightly less (*r*^*2*^ ~ 0.3) and MK (downsampled to 10 individuals; see “Methods”) exhibited an LD decay similar to wild (*r*^*2*^ ~ 0.2).

### Number of novel alleles

**Figure 4:**
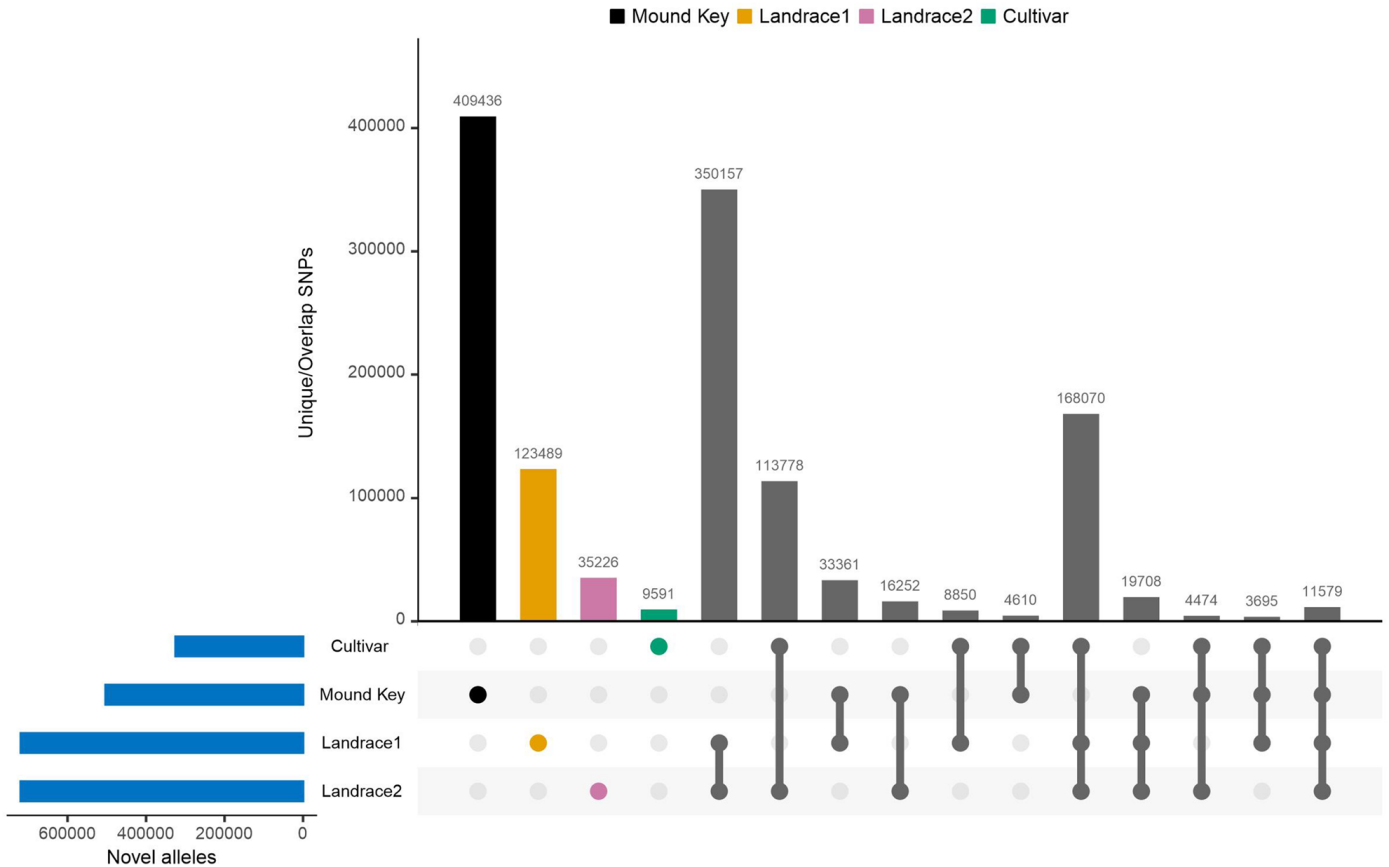
Distribution of alleles (SNPs) found in Mound Key, landrace1, landrace2, and cultivar that were not observed in the wild cottons. The total number of non-wild (novel) allele sites for each comparison is represented by the blue horizontal barplot on the bottom left. The number of sites unique among each group is represented by a colored dot and a vertical barplot on the right side (first four dots and vertical bars). The following dots and bins represent the number of sites that are shared among groups, which are indicated by a line joining two, three, or four dots under the bar plot.

Given the geographical isolation of Mound Key from previously collected wild *G. hirsutum* (from Yucatan to Caribbean), we evaluated the MK cottons for evidence of ‘novel’ genotypes, either fixed or segregating. We also tabulated the non-wild alleles in three domesticated groups that may result from domestication or genome introgression from sister species (see “Discussion”). Out of 12.2 M filtered SNP sites across all 67 samples (65 *G. hirsutum* and two *G. mustelinum*), 1.3 M (10.8%) sites from MK and the three domesticated groups had genotypes that were not observed in the wild cottons or the outgroup, indicating they may be derived or originate from previously unsampled diversity. Specifically, both landraces groups exhibited a similar percentage (*c.* 5.9%), followed by MK (4.1%), and then cultivars (2.7%; Fig. 4).

We parsed the SNP sites according to whether they are unique to one group or are overlapped between groups (Fig. 4). All three domesticated groups exhibited large overlaps in the SNP sites not found in the wild (350,157 present in both landraces; 113,778 in LR2 and cultivars; 8850 in LR1 and cultivar; and 168,070 in all three). Interestingly, the MK cotton exhibited the greatest number of unique SNP sites (409,436), and followed by higher unique SNP sites in LR1 (123,489) than LR2 and cultivar (35,226 and 9591, respectively).

### Diversity within Mound Key Cotton

To further understand the genetic variation of the Mound Key population, we filtered 5,840,480 SNPs to include only the 25 MK individuals. The proportion of heterozygosity sites among MK filtered SNPs ranged from 32 to 50%, with an average around 40%. With respect to the genomic distribution of heterozygosity, the At subgenome contained twice as many filtered SNPs (3,974,187 vs 1,866,293) resulting in higher proportion of heterozygous sites (27% vs 13%, out of total 5.8 M sites) than the Dt subgenome.

**Figure 5:**
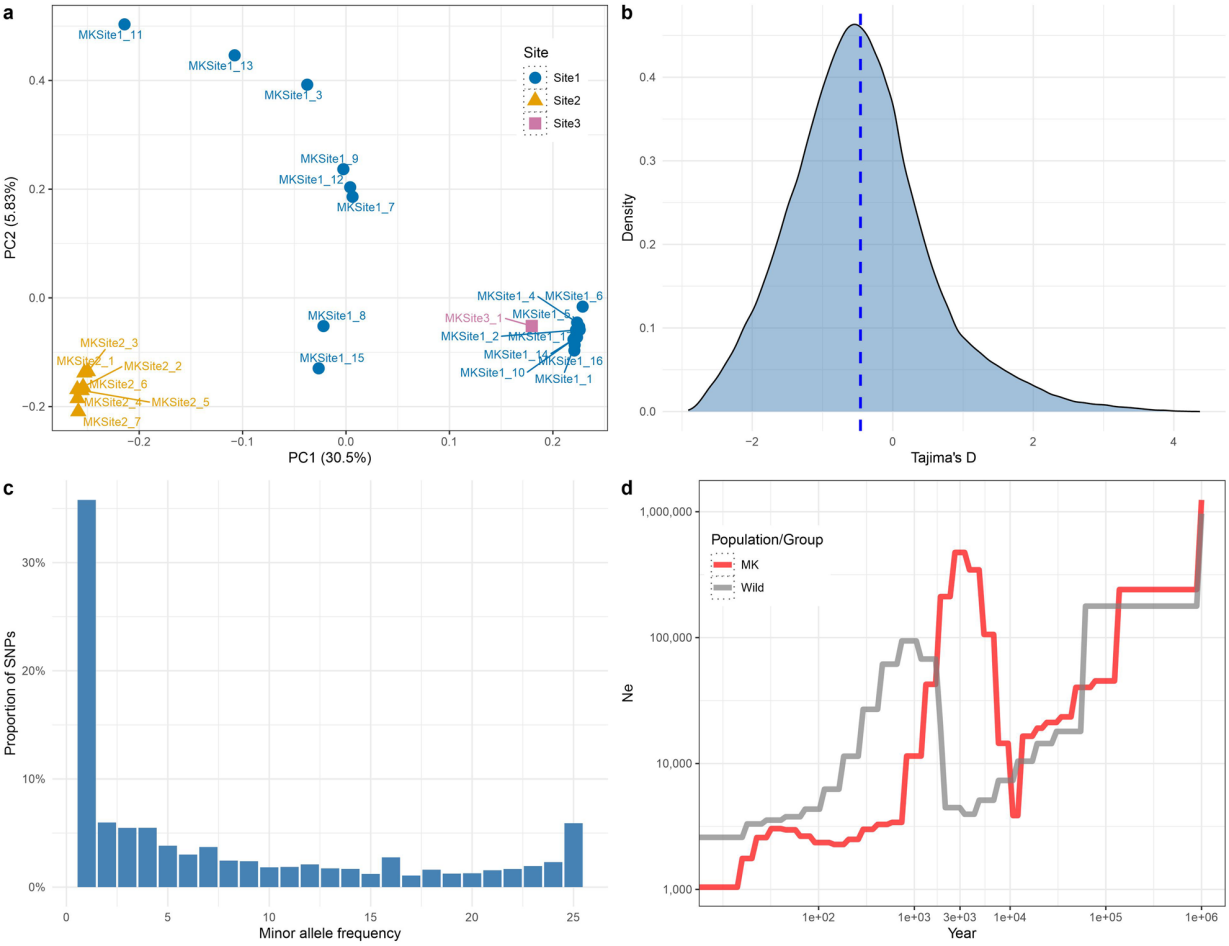
Variation and evolutionary histories of Mound Key (MK) cotton. (**a**) PCA variation of MK population where each site is labeled by distinguished color and shape. (**b**) Density plot of genome-wide Tajima’s D across a window size of 50 kbp. (**c**) Folded site frequency spectrum using minor allele frequency for Mound Key cotton. (**d**) Population demographic analysis for Mound Key population and wild group reconstructing the changes in effective population size (*Ne*) along evolutionary time.

A PCA using filtered SNPs (16,005 unlinked genic sites), with PC1 explaining 30.5% of total variation, partitioned the MK samples into two groups and a few scattered individuals (Fig. 5a). Specifically, the first group (right side of PCA) contained nine Site1 individuals and the sole Site3 individual. The second group containing seven individuals from Site2 (left side of PCA) exhibited a similar degree of intra-group divergence to the first group. The remaining eight individuals from Site1 were scattered in the middle of the PCA. The groups also supported population LEA structure using just the MK population (Fig. S3). Samples within each group also showed high kinships in terms of genetic relatedness (Table S3).

On average, Mound Key cotton exhibited a negative Tajima’s D (-0.46; Fig. 5b), suggesting an excess of rare variants, which was reiterated in the shape of the site frequency spectrum (SFS; Fig. 5c). Accordingly, population demography analysis for Mound Key (as well as for all wild accessions) suggests a series of population crashes and recoveries, including an inferred expansion *c.* 3000 years ago (ya) that followed a bottleneck *c.* 10,000 ya (Fig. 5d). As expected from the influence of human activities, the effective population size exhibits a declining trend from 100 to 1000 ya to the present.

## Discussion

Wild relatives represent important genomic resources for agricultural improvement. Although domesticated cultivars of *G. hirsutum* contribute to more than 90% of world-wide cotton production^18^, little is known about genetic diversity within the wild forms. As with most domesticated species, such as rice^19^ or maize^20^, the consequences of human-mediated directional selection for desired traits also winnows pre-existing genetic diversity in *G. hirsutum*, as demonstrated by allozymes^9^, anonymous molecular markers^8^, and SNP data derived from whole genome resequencing^15^. Compared to traditional morphological and/or geographical based wild cotton identification^6,11,21,22^, genome-wide SNP data^15^ in particular provide a powerful tool for resolving questions about the origin, diversification and domestication history of *G. hirsutum*.

Yuan et al.^15^ compared genomic diversity and genetic relationships of globally sampled *G. hirsutum* cultivars and a broad sampling of wild and feral samples, leading to the categorization of the species into four major groups with three different levels of genetic diversity. Using a subset of sampling from these four groups, our results showed similar relationships within and between these four groups (Fig. 2; Table 2). The two landrace groups (LR1 and LR2) were similar to early domesticated forms, which may have originated along the northern coastal regions of the Yucatan Peninsula in Mexico^6,8,9^, from where they spread south through the Peninsula into central Mexico and then later throughout Mesoamerica and the Caribbean basin in pre-colonial times^6,22^. By contrast, a more severe loss of diversity is evident in the cultivar group, most likely originating from landrace2 (Fig. 2b), which went through sequential additional bottlenecks since European colonization of the Americas.

Mound Key, Florida (USA) is located in Estero Bay along the SW coastline, in a region in which there are many historical and contemporary reports of remnant wild populations of *G. hirsutum*^12,13,23^. In the wild, *G. hirsutum* occupies littoral habitats, which provide opportunities for seed dispersal and population establishment to remote regions sometimes separated by water bodies. The population from Mound Key is thus ecologically consistent with it being wild as opposed to having been derived from a previous stage of domestication.

This ecological evidence is bolstered by the genetic data; compared to the three domesticated groups (LR1, LR2 and cultivars), each with complex histories that include bottlenecks associated with selection and human dispersal, as well as genome introgression from a second cultivated cotton species, *G. barbadense*^15,24,25^, the Mound Key population exhibited the highest Fst genetic divergence from the three domesticated groups, even higher than that of the wild group (Fig. 3d). Moreover, we note that although total diversity within the Mound Key population, as measured by nucleotide diversity (Table 2; Fig, 3a), is about half of that exhibited by the wild cottons as a whole, the Mound Key population has low dxy nucleotide divergence from the wild group compared to wild vs the three domesticated groups (Fig. 3c), further bolstering the interpretation that the Mound Key population may truly be wild.

Complementing the inference derived from genetic distance and composition, the wild and Mound Key cottons have similar LD decay profile (Fig. 3b), and therefore likely share multiple population-level features reviewed in^26^, such as relatively small effective population sizes, clustered population structure, and both generalized and actual inbreeding. Importantly, the Mound Key population is not embedded within the broader wild group, but instead, is distinct from it in the genetic space (Fig. 2). We conclude that the MK population not only is wild, but that it represents a gene pool that was previously unrecognized. We infer that diversity within truly wild *G. hirsutum* may be much greater than previously documented, calling for further sampling in and around the Caribbean region.

From a quantitative perspective, wild populations are expected to harbor greater diversity than domesticated conspecific gene pools. Similarly, escaped, feral derivatives are expected to contain only a fraction of the diversity of their progenitor lineage, and with little time for the evolution of new nucleotide diversity (here, alleles) to emerge. This is reflected here in the comparative analysis of novel alleles (Fig. 4). The Mound Key population exhibits what we consider to be a large number of new alleles relative to the previously recognized wild group (Fig. 4). This is especially impressive when one considers that the Mound Key cottons were sampled within about a 400 m transect from one tiny island off the coast of SW Florida. By extension, it is reasonable to suggest, therefore, that the total diversity contained within wild *G. hirsutum* was once much greater than recorded to date, and that many other pockets of unrecognized diversity within its distribution range likely remain to be discovered.

Available historical, genetic, and ecological evidence indicates that wild *G. hirsutum* populations with multiple geographically widely dispersed and highly localized pools of genetic diversity, and possibly that much of the diversity would have been novel with respect to other populations. Habitat restriction to coastal environments with a fluctuating sealine over multiple glacial cycles^27^ creates a shifting distribution of restricted and ephemeral populations, a situation exacerbated by localized extinctions due to tropical storms and hurricanes, and more recently, by massive habitat loss due to human activity. These dynamics create a highly fragmented wild gene pool, both in time and in space, with a shifting metapopulation comprising numerous geographically small and disconnected subpopulations^12^. In this respect, we note that the inferred population demographic of Mound Key showed a large bottleneck at 10,000 ya (Fig. 4D) when the sea level started to rise, submerging the Florida coastline^28^. In addition to these ecological and glacial history considerations, *G. hirsutum* contains a suite of life-history features that constrain diversity, including both generalized and actual inbreeding and relatively small population sizes.

A main finding emerges from the present study, namely, that even sampling from one tiny island near the margin of the wild range of *G. hirsutum* reveals a pocket of unsuspected and apparently novel diversity. This seems extraordinary given the importance of the species as the source of the world’s most economically important cotton crop plant. Similar novel diversity patterns have also been found in non-crop relictual populations of *Arabidopsis thaliana*^29^, *Pinus bungeana *^30^, *Taxus baccata *^31^. To better understand wild *G. hirsutum* diversity, it is necessary to sample more broadly throughout the geographical range, including but not limited to the putative ancestral Yucatan region of Mexico. The information gleaned from such analyses may help identify genomic sources of adaptive phenotypes, including, for example, salt tolerance^32^. Finally, the present study serves as a reminder of the importance of reserves such as Mound Key, Florida, which is a State of Florida Archaeological Park and is thus protected from development. Understanding genetic diversity within wild *G. hirsutum* is crucial for future genomic diversity conservation.

## Methods

### Sample collection, DNA extraction & sequencing

Leaves from 25 Mound Key individuals were collected from three sites and preserved in silica gel (Fig. 1). All collection activities were completed under Florida Park Service research and collection permit numbers 10312110, 10312110A, and 02092414. The vouchers of specimens (Fig. 1a) were pressed and deposited in Ada Hayden Herbarium (ISC) inside Bessey Hall at Iowa State University.

The dry cotton leaf tissues (about 30 to 50 mg) were disrupted into fine powders with 2.0-mm ZR Bashing Beads (Zymo Research, Irvine, CA, USA) using the Geno/Grinder 2010 system (SPEX SamplePrep, Metuchen, NJ, USA) at 1500 rpm for 30 s. The genomic DNA was extracted from cotton leaf powders using Qiagen Plant DNeasy Mini kit (Qiagen, Germantown, MD, USA) following the manufacturer’s instruction. The concentration and purity of extracted genomic DNA were measured by the NanoDrop One spectrophotometer (Thermofisher Scientific, Waltham, MA, USA). The genomic DNA integrity was validated by agarose gel electrophoresis.

A total of 300 mg to 500 mg of genomic DNA from each cotton sample was subjected to the Illumina DNA-Seq library preparation using NEBNext Ultra II FS DNA Library Prep Kit and NEBNext Multiplex Dual Index Primers Set (New England Biolabs, Ipswich, MA, USA) based on the manufacturer’s manuals. Genomic libraries were equimolarly pooled together and sequenced on one lane of NovaSeq S4 system (Illumina, San Diego, CA) with paired-ended 150 bp sequencing length by Novogene Corporation (https://www.novogene.com/us-en/).

Published whole genome resequencing data were retrieved for 10 individuals from each of the four predefined groups (wild, landrace1, landrace2, and cultivars), and two *G. mustelinum* individuals (genome designation AD_4_) as outgroup (Table S1). Selection criteria for these individuals were based on sequencing depth > 20x (if known) and their representative distribution within the phylogenetic framework and principal component analyses (PCA) of the broad germplasm pool (Fig. S1; Table S1)^15^.

### Raw read trimming, mapping, & variant calling

Raw reads for all 65 samples were trimmed using Trimmomatic V.0.39^33^ with ‘ILLUMINACLIP:Adapters.fa:2:30:10:2:True LEADING:3 TRAILING:3 MINLEN:75’ settings to remove adaptors and low quality bases. Trimmed reads were then grouped (-R) and mapped to the reference genome of a wild *G. hirsutum* accession TX2094 v1.0 (unpub. data) via BWA^34^. Genome wide variations including SNPs and small insertion and deletion (indels) were identified for each individual using the Sentieon DNASeq variant-calling pipeline^35^, which offers a less computationally expensive method, highly similar to the GATK best-practices workflow^36^.

Sentieon involved first sorting mapped reads and converting output to the bam file format (‘–sam2bam’), marking duplicated reads (using flags ‘–algo LocusCollector’ and ‘–algo Dedup’) and removing duplicates (‘–rmdup’). Following indel realignment (‘–algo realigner’), coverage depths were caculated (‘–algo CoverageMetrics’) using the final sorted bam files. Variants were called (‘–algo Haplotyper’ and ‘–emit_mode gvcf’) for each sample (i.e., as the gVCF file), producing a master VCF file through joint-calling using the gVCF files from all samples (‘–algo GVCFtyper’ and ‘–emit_mode all’), which contain both variable (SNP and indels) and invariable sites (i.e., consistent sites that match the reference genotype) (details of the protocol: https://github.com/Wendellab/MoundKeyCottons). Using vcftools^37^, the master VCF was divided into two files: the invariable-VCF (‘–max-maf 0’) and a filtered SNP-VCF (‘–max-missing-count 0 –max-alleles 2 –mac 1 –maf 0.05’), respectively; all the indels were removed (‘–remove-indels’). and positions were filtered by average minimal depth less than 10 (‘–min-meanDP 10’) or above 100 (‘–max-meanDP 100’) in both VCFs. Finally, the two filtered VCF files were combined (flag ‘concat’) by bcftools^38^, hereafter combined-VCF, for downstream analysis (https://github.com/Wendellab/MoundKeyCottons).

A separate combined-VCF for additional two outgroup *G. mustelinum* individuals (AD_4_) was also reconstructed using the same settings as above. We merged two combined-VCF files for 65 samples and two AD_4_ samples via bcftools (‘merge’), and extracted only biallelic SNPs for across all 67 samples for later phylogenetic analysis and novel allele counting.

### Population genetic relationships

From the combined-VCF of all 65 AD1 samples, only their genic SNPs were extracted by bedops^39^ and vcftool (–bed), based on the annotated gene models of the reference genome TX2094 v1.0 (unpub. data). Using PLINK v.1.9^40^, any genic SNPs with missing data were removed (‘–geno 0’), and LD between SNPs was pruned with ‘–indep-pairwise 50 10 0.1’ (https://github.com/Wendellab/MoundKeyCottons).

The unlinked genic SNPs (11,320; see “Results”) were used to conduct PCA analysis in PLINK (–PCA 20). The population genetic structure between MK and the four *G. hirsutum* groups was estimated using LEA^41^ from the same set of SNPs. The population number K (ancestral populations) was tested from 1 to 15, running each K configuration 10 times. The K value with the lowest average cross-entropy from those 10 runs was selected as the best model.

To generate a rooted phylogenetic tree, using similar filtering steps and the same annotation file as above, we extracted 10,322 LD pruned genic SNPs from the combined-VCF for 67 samples via vcftools (‘–bed’; ‘–max-missing-count 0 –max-alleles 2 –min-meanDP 10 –max-meanDP 100 –mac 1 –maf 0.05’) and PLINK (‘–indep-pairwise 50 10 0.1’). The genetic distance matrix was calculated via PLINK (–distance square 1-ibs), and we reconstructed the phylogenetic relationships using the neighbor-joining method via R package APE^42^ and visualized by package ggtree^43^.

### Genetic diversity analysis

The proportion of heterozygosity were calculated among all filtered SNPs (7,580,890; see “Results”) to estimate the Wright's inbreeding coefficient F_IS_, using vcftools (–het). Genetic linkage disequilibrium (LD) for MK and each group was estimated via PopLDdecay^44^ by calculating the squared correlation (*r*^*2*^) between all pairs of SNPs within 500 kbp windows (-MaxDist 500). To minimize LD estimation biases due to sample size variations, we randomly down-sampled 25 MK individuals to 10 individuals from filtered SNPs and repeated this process 10 times for comparison across groups.

Genome-wide nucleotide diversities (π) within MK and all four groups were estimated using the combined-VCF (including both variable SNPs and invariable sites) via Pixy^45^ with a sliding-window size of 10 kbp. Pairwise differences of sequence diversity (dxy) and population genetic differentiation (Fst) were also calculated in Pixy using the combined-VCF with a sliding-window size of 10 kbp between groups.

### Novel allele identification

Compared to the genotypes in the wild samples, the appearance of novel alleles (i.e. SNPs) within and among MK, LR1, LR2, and cultivar may also provide insight into their origins and divergence, reflecting domestication/bottleneck or genomic introgression events (see “Results”). An allele was considered novel if at a given SNP site, the genotypes of all 10 wild individuals and two outgroup AD_4_ individuals are homozygous for the reference (i.e., ‘0/0’ in VCF file) and at least one individual in the other groups had an alternative genotype (i.e., ‘0/1’ or ‘1/1’). Using the combined-VCF for all 67 samples, we filtered only biallelic SNPs via vcftools (‘–max-missing-count 0 –mac 1 –max-alleles 2’). We identified the number of SNPs for each group (i.e., MK, LR1, LR2, and cultivar) that contained newly derived alleles from the total of 12,190,541 filtered SNPs (see “Results”). The overlap and unique SNPs (the ‘ID’ column in VCF) among the different groups were plotted using UpSetR^46^ in R.

### Variation within Mound Key Cotton

To investigate sub-population structure within the MK samples, we extracted SNPs using the gVCFs of only 25 MK individuals. SNPs were filtered with the same thresholds noted above for minor allele frequency and average depth. Using all filtered SNPs (5,840,480) for MK samples only, we estimated the number of heterozygous sites for each individual (‘–het’) and calculated the kinships (‘–relatedness’)^47^ between those 25 individuals via vcftools (https://github.com/Wendellab/MoundKeyCottons).

After pruning the LD among the filtered SNPs (16,005) for only MK samples via PLINK ‘–indep-pairwise 50 10 0.1’, using the same steps as above, we also assessed the subpopulation structure within Mound Key using PCA, neighbor-joining tree, and via LEA genetic structure (https://github.com/Wendellab/MoundKeyCottons).

The folded site frequency spectrum (SFS) was also estimated using the final sorted bam file from 25 individuals by ANGSD and realSFS^48^ (with following settings ‘-doSaf 1 -doMaf 1 -doMajorMinor 1 -doGlf 3 -uniqueOnly -GL 2 -minMapQ 30 -minQ 20 -minInd 25’). We also calculated the genome-wide Tajima’s D^49^ by ANGSD and thetastat (‘-win 50,000 -step 10,000’). The population demographic history for MK (changes in effective population size N_e_ over evolutionary time) was modeled in SMC++^50^ using all filtered SNPs as input, by specifying time points from 10 to 1,000,000 generations, mutation rate as 4.56e-9^51,52^, and a 1-year of generation time. To compare with the wild cotton N_e_ change, we performed the SMC++ estimation for 10 wild individuals plus one MK individual (MKSite1_10) using 7,580,890 SNPs extracted from all 65 AD1 SNP-VCF.

### Supplementary Information

Supplementary Information.

## Data Availability

Bioinformatic pipelines are uploaded to: https://github.com/Wendellab/MoundKeyCottons; All sequence data generated in this study are available at the NCBI (PRJNA1086501).
