# Supplementary material for: Origin and diversity of the wild cottons (Gossypium hirsutum) of Mound Key, Florida
Source: Sci Rep. 2024 Jun 18;14:14046. doi: 10.1038/s41598-024-64887-8 (PMC11189519; doi:10.1038/s41598-024-64887-8)
Supplement: Supplementary file 1 — Supplementary Information. [file 41598_2024_64887_MOESM1_ESM.pdf]

Supplementary

Title: Origin and diversity of the wild cottons (*Gossypium hirsutum*) of Mound Key, Florida

Author list: Weixuan Ning[1], Karen M. Rogers[2], Chuan-Yu Hsu[3], Zenaida V Magbanua[3], Olga Pechanova[3], Mark A Arick II[3], Ehsan Kayal[1], Guanjing Hu[4,5], Daniel G Peterson[3], Joshua A Udall[6], Corrinne E Grover[1*], Jonathan F Wendel[1*]

[1] Ecology, Evolution, and Organismal Biology Dept., Iowa State University, Ames, IA, 50011, USA

[2] Florida Department of Environmental Protection, Division of Recreation and Parks, District 4 Administration, 1843 S. Tamiami Trail, Osprey, FL 34229, USA.

[3] Institute for Genomics, Biocomputing & Biotechnology, Mississippi State University, Mississippi State, MS 39762, USA

[4] National Key Laboratory of Cotton Bio-breeding and Integrated Utilization, Institute of Cotton Research, Chinese Academy of Agricultural Sciences, Anyang, 455000, China

[5] Shenzhen Branch, Guangdong Laboratory of Lingnan Modern Agriculture, Key Laboratory of Synthetic Biology, Ministry of Agriculture and Rural Affairs, Agricultural Genomics Institute at Shenzhen, Chinese Academy of Agricultural Sciences, Shenzhen, 518120, China

[6] Crop Germplasm Research Unit, USDA/Agricultural Research Service, 2881 F&B Road, College Station, TX 77845, USA

* these authors contributed equally; * corresponding author email: jfw@iastate.edu

Table S1. Sampling information for Mound Key, four predesignated groups (landrace1, landrace2, cultivar and wild) [15] and two AD_4 individuals (outgroup). The average coverage (if known) and sequence reads data (SRR) for each individual are also listed.

Population/Group	Individual	Average coverage	SRR
Cultivar	Cultivar_B11SA0825	22.36	SRR6311822
Cultivar	Cultivar_B11SA1427	23.63	SRR6311783
Cultivar	Cultivar_B11SA1465	25.66	SRR6311779
Cultivar	Cultivar_P115HAU041		SRR4006675
Cultivar	Cultivar_P115HAU059		SRR4006691
Cultivar	Cultivar_P115HAU066		SRR4006698
Cultivar	Cultivar_P115HAU094		SRR4006732
Cultivar	Cultivar_P115HAU101		SRR4013321
Cultivar	Cultivar_P115HAU146		SRR4013897
Cultivar	Cultivar_P115HAU242		SRR4018588
Landrace1	Landrace1_B12BPS1238	22.09	SRR6311488
Landrace1	Landrace1_B12TX1578	24.08	SRR6311813
Landrace1	Landrace1_B12TX1592	29.38	SRR6311758
Landrace1	Landrace1_B12TX1689	21.87	SRR6311762
Landrace1	Landrace1_B12TX1935	24.17	SRR6311799
Landrace1	Landrace1_B12TX2216	25.27	SRR6311806
Landrace1	Landrace1_B12TX2226	21.54	SRR6311807
Landrace1	Landrace1_B12TX2465	27.46	SRR6311600
Landrace1	Landrace1_B12TX2487	25.07	SRR6311752
Landrace1	Landrace1_B12TX2489	23.18	SRR6311749
Landrace2	Landrace2_B12TX0109	23.95	SRR6311544
Landrace2	Landrace2_B12TX0240	26.52	SRR6311653
Landrace2	Landrace2_B12TX0390	24.86	SRR6311805
Landrace2	Landrace2_B12TX0487	27.33	SRR6311550
Landrace2	Landrace2_B12TX1102	21.81	SRR6311801
Landrace2	Landrace2_B12TX1111	25.68	SRR6311759
Landrace2	Landrace2_B12TX1459	22.93	SRR6311768
Landrace2	Landrace2_B12TX1661	25.49	SRR6311747
Landrace2	Landrace2_B12TX1721	20.34	SRR6311751
Landrace2	Landrace2_B12TX1981	24.3	SRR6311802
Mound Key	MKSite1_1	30.15	SRR28292574
Mound Key	MKSite1_10	24.28	SRR28292550
Mound Key	MKSite1_11	25.97	SRR28292570
Mound Key	MKSite1_12	22.87	SRR28292569
Mound Key	MKSite1_13	19.55	SRR28292571
Mound Key	MKSite1_14	24.9	SRR28292568
Mound Key	MKSite1_15	19.26	SRR28292572

Mound Key	MKSite1_16	19.34	SRR28292567
Mound Key	MKSite1_17	24.74	SRR28292566
Mound Key	MKSite1_2	24.23	SRR28292573
Mound Key	MKSite1_3	20.84	SRR28292561
Mound Key	MKSite1_4	27.95	SRR28292556
Mound Key	MKSite1_5	24.48	SRR28292555
Mound Key	MKSite1_6	24.26	SRR28292554
Mound Key	MKSite1_7	26.74	SRR28292553
Mound Key	MKSite1_8	20.82	SRR28292552
Mound Key	MKSite1_9	21.05	SRR28292551
Mound Key	MKSite2_1	24.92	SRR28292563
Mound Key	MKSite2_2	26.48	SRR28292562
Mound Key	MKSite2_3	25.64	SRR28292564
Mound Key	MKSite2_4	23.27	SRR28292565
Mound Key	MKSite2_5	23.61	SRR28292560
Mound Key	MKSite2_6	25.76	SRR28292559
Mound Key	MKSite2_7	18.57	SRR28292558
Mound Key	MKSite3_1	21.53	SRR28292557
Wild	Wild_B12BPS1151	23.09	SRR6311622
Wild	Wild_B12BPS1244	23.96	SRR6311625
Wild	Wild_B12BPS1247	20.17	SRR6311626
Wild	Wild_B12TX0967	28.62	SRR6311726
Wild	Wild_B12TX1894	20.63	SRR6311795
Wild	Wild_B12TX1996	27.19	SRR6311499
Wild	Wild_B12TX2090	21.32	SRR6311615
Wild	Wild_B12TX2211	21.65	SRR6311787
Wild	Wild_B12TX2276	21.12	SRR6311607
Wild	Wild_P121TX2094		SRR1975549
AD_4	AD4_mus_P401AD4	21.96	SRR1975566
AD_4	AD4_mus_B40AD4W		SRR6334671

Table S2. Observed homozygosity (Ob_Ho), observed heterozygosity (Ob_He), expected homozygosity (Exp_Ho), and percentage Ob_He (%), using 7,580,890 filtered SNPs for each individual. Wright's inbreeding coefficient (F_{IS}) also calculated for each sample.

Individual	Ob_Ho	Ob_He	Exp_Ho	Total	Ob_He (%)	FIS
Cultivar_B11SA0825	7,076,416	504,474	5,107,559	7,580,890	0.07	0.8
Cultivar_B11SA1427	7,185,131	395,759	5,107,559	7,580,890	0.05	0.84
Cultivar_B11SA1465	7,118,442	462,448	5,107,559	7,580,890	0.06	0.81
Cultivar_P115HAU041	7,385,196	195,694	5,107,559	7,580,890	0.03	0.92
Cultivar_P115HAU059	7,374,242	206,648	5,107,559	7,580,890	0.03	0.92
Cultivar_P115HAU066	7,352,621	228,269	5,107,559	7,580,890	0.03	0.91
Cultivar_P115HAU094	7,332,562	248,328	5,107,559	7,580,890	0.03	0.9
Cultivar_P115HAU101	7,405,400	175,490	5,107,559	7,580,890	0.02	0.93
Cultivar_P115HAU146	7,231,655	349,235	5,107,559	7,580,890	0.05	0.86
Cultivar_P115HAU242	7,382,020	198,870	5,107,559	7,580,890	0.03	0.92
Landrace1_B12BPS1238	4,520,353	3,060,537	5,107,559	7,580,890	0.4	-0.24
Landrace1_B12TX1578	6,868,810	712,080	5,107,559	7,580,890	0.09	0.71
Landrace1_B12TX1592	6,926,677	654,213	5,107,559	7,580,890	0.09	0.74
Landrace1_B12TX1689	6,923,094	657,796	5,107,559	7,580,890	0.09	0.73
Landrace1_B12TX1935	6,926,261	654,629	5,107,559	7,580,890	0.09	0.74
Landrace1_B12TX2216	6,796,018	784,872	5,107,559	7,580,890	0.1	0.68
Landrace1_B12TX2226	6,886,500	694,390	5,107,559	7,580,890	0.09	0.72
Landrace1_B12TX2465	6,590,856	990,034	5,107,559	7,580,890	0.13	0.6
Landrace1_B12TX2487	6,786,331	794,559	5,107,559	7,580,890	0.1	0.68
Landrace1_B12TX2489	6,955,172	625,718	5,107,559	7,580,890	0.08	0.75
Landrace2_B12TX0109	7,199,472	381,418	5,107,559	7,580,890	0.05	0.85
Landrace2_B12TX0240	7,539,168	41,722	5,107,559	7,580,890	0.01	0.98
Landrace2_B12TX0390	6,892,211	688,679	5,107,559	7,580,890	0.09	0.72
Landrace2_B12TX0487	7,119,164	461,726	5,107,559	7,580,890	0.06	0.81
Landrace2_B12TX1102	7,135,244	445,646	5,107,559	7,580,890	0.06	0.82
Landrace2_B12TX1111	7,142,250	438,640	5,107,559	7,580,890	0.06	0.82
Landrace2_B12TX1459	7,042,998	537,892	5,107,559	7,580,890	0.07	0.78
Landrace2_B12TX1661	6,500,670	1,080,220	5,107,559	7,580,890	0.14	0.56
Landrace2_B12TX1721	6,830,502	750,388	5,107,559	7,580,890	0.1	0.7
Landrace2_B12TX1981	7,054,654	526,236	5,107,559	7,580,890	0.07	0.79
MKSite1_1	6,626,563	954,327	5,107,559	7,580,890	0.13	0.61
MKSite1_10	6,655,705	925,185	5,107,559	7,580,890	0.12	0.63
MKSite1_11	6,660,385	920,505	5,107,559	7,580,890	0.12	0.63
MKSite1_12	6,412,894	1,167,996	5,107,559	7,580,890	0.15	0.53
MKSite1_13	6,543,300	1,037,590	5,107,559	7,580,890	0.14	0.58
MKSite1_14	6,662,610	918,280	5,107,559	7,580,890	0.12	0.63
MKSite1_15	6,366,307	1,214,583	5,107,559	7,580,890	0.16	0.51

MKSite1_16	6,690,997	889,893	5,107,559	7,580,890	0.12	0.64
MKSite1_17	6,659,752	921,138	5,107,559	7,580,890	0.12	0.63
MKSite1_2	6,662,894	917,996	5,107,559	7,580,890	0.12	0.63
MKSite1_3	6,532,988	1,047,902	5,107,559	7,580,890	0.14	0.58
MKSite1_4	6,642,427	938,463	5,107,559	7,580,890	0.12	0.62
MKSite1_5	6,708,683	872,207	5,107,559	7,580,890	0.12	0.65
MKSite1_6	6,766,515	814,375	5,107,559	7,580,890	0.11	0.67
MKSite1_7	6,378,472	1,202,418	5,107,559	7,580,890	0.16	0.51
MKSite1_8	6,357,972	1,222,918	5,107,559	7,580,890	0.16	0.51
MKSite1_9	6,554,800	1,026,090	5,107,559	7,580,890	0.14	0.59
MKSite2_1	6,610,859	970,031	5,107,559	7,580,890	0.13	0.61
MKSite2_2	6,566,662	1,014,228	5,107,559	7,580,890	0.13	0.59
MKSite2_3	6,605,801	975,089	5,107,559	7,580,890	0.13	0.61
MKSite2_4	6,600,445	980,445	5,107,559	7,580,890	0.13	0.6
MKSite2_5	6,581,048	999,842	5,107,559	7,580,890	0.13	0.6
MKSite2_6	6,594,790	986,100	5,107,559	7,580,890	0.13	0.6
MKSite2_7	6,635,762	945,128	5,107,559	7,580,890	0.12	0.62
MKSite3_1	6,555,866	1,025,024	5,107,559	7,580,890	0.14	0.59
Wild_B12BPS1151	6,824,699	756,191	5,107,559	7,580,890	0.1	0.69
Wild_B12BPS1244	6,662,645	918,245	5,107,559	7,580,890	0.12	0.63
Wild_B12BPS1247	5,296,649	2,284,241	5,107,559	7,580,890	0.3	0.08
Wild_B12TX0967	6,107,336	1,473,554	5,107,559	7,580,890	0.19	0.4
Wild_B12TX1894	6,635,222	945,668	5,107,559	7,580,890	0.12	0.62
Wild_B12TX1996	6,837,846	743,044	5,107,559	7,580,890	0.1	0.7
Wild_B12TX2090	6,975,741	605,149	5,107,559	7,580,890	0.08	0.76
Wild_B12TX2211	6,490,245	1,090,645	5,107,559	7,580,890	0.14	0.56
Wild_B12TX2276	6,805,323	775,567	5,107,559	7,580,890	0.1	0.69
Wild_P121TX2094	6,279,258	1,301,632	5,107,559	7,580,890	0.17	0.47

Table S3. Genetic relatedness [43] between 25 Mound Key individuals. Only kinship shows the “twins” (0.35 to 0.5) or ‘1st-degree’ (0.178 to 0.35) levels are shown in the table.

	MKSite1_1	MKSite1_10	MKSite1_11	MKSite1_12	MKSite1_13	MKSite1_14	MKSite1_15	MKSite1_16	MKSite1_17	MKSite1_2	MKSite1_3	MKSite1_4	MKSite1_5	MKSite1_6	MKSite1_7	MKSite1_8	MKSite1_9	MKSite2_1	MKSite2_2	MKSite2_3	MKSite2_4	MKSite2_5	MKSite2_6	MKSite2_7
MKSite1_10	0.33																							
MKSite1_11	0	0																						
MKSite1_12	0.27	0.27	0.27																					
MKSite1_13	0	0	0.29	0.32																				
MKSite1_14	0.33	0.33	0	0.27	0																			
MKSite1_15	0.26	0.26	0.19	0.32	0.27	0.26																		
MKSite1_16	0.32	0.32	0	0.27	0	0.32	0.25																	
MKSite1_17	0.33	0.33	0	0.27	0	0.33	0.26	0.32																
MKSite1_2	0.33	0.33	0	0.27	0	0.33	0.26	0.32	0.33															
MKSite1_3	0.21	0.21	0.24	0.32	0.29	0.21	0.28	0.2	0.21	0.21														
MKSite1_4	0.34	0.33	0	0.27	0	0.33	0.26	0.32	0.33	0.33	0.21													
MKSite1_5	0.33	0.33	0	0.27	0	0.33	0.25	0.31	0.33	0.32	0.2	0.33												
MKSite1_6	0.32	0.32	0	0.27	0	0.32	0.24	0.31	0.32	0.32	0.19	0.32	0.33											
MKSite1_7	0.28	0.28	0.28	0.37	0.33	0.28	0.32	0.27	0.28	0.28	0.33	0.28	0.28	0.27										
MKSite1_8	0.27	0.26	0.2	0.33	0.27	0.26	0.35	0.26	0.26	0.26	0.28	0.27	0.26	0.26	0.33									
MKSite1_9	0.21	0.21	0.2	0.31	0.25	0.21	0.27	0.2	0.2	0.2	0.28	0.21	0.2	0.19	0.31	0.28								
MKSite2_1	0	0	0	0	0	0	0.2	0	0	0	0	0	0	0	0	0.2	0							
MKSite2_2	0	0	0	0.19	0	0	0.25	0	0	0	0	0	0	0	0.2	0.26	0	0.22						
MKSite2_3	0	0	0	0	0	0	0.2	0	0	0	0	0	0	0	0	0.2	0	0.34	0.22					
MKSite2_4	0	0	0	0	0	0	0.27	0	0	0	0	0	0	0	0.18	0.27	0	0.19	0.3	0.2				
MKSite2_5	0	0	0	0.19	0	0	0.26	0	0	0	0	0	0	0	0.19	0.27	0	0.19	0.3	0.19	0.31			
MKSite2_6	0	0	0	0	0	0	0.24	0	0	0	0	0	0	0	0.18	0.24	0	0.19	0.29	0.2	0.28	0.31		
MKSite2_7	0	0	0	0	0	0	0.25	0	0	0	0	0	0	0	0	0.25	0	0.19	0.31	0.19	0.31	0.3	0.26	
MKSite3_1	0.31	0.31	0	0.29	0.19	0.31	0.28	0.3	0.31	0.31	0.23	0.31	0.3	0.29	0.29	0.28	0.24	0	0	0	0	0	0	0

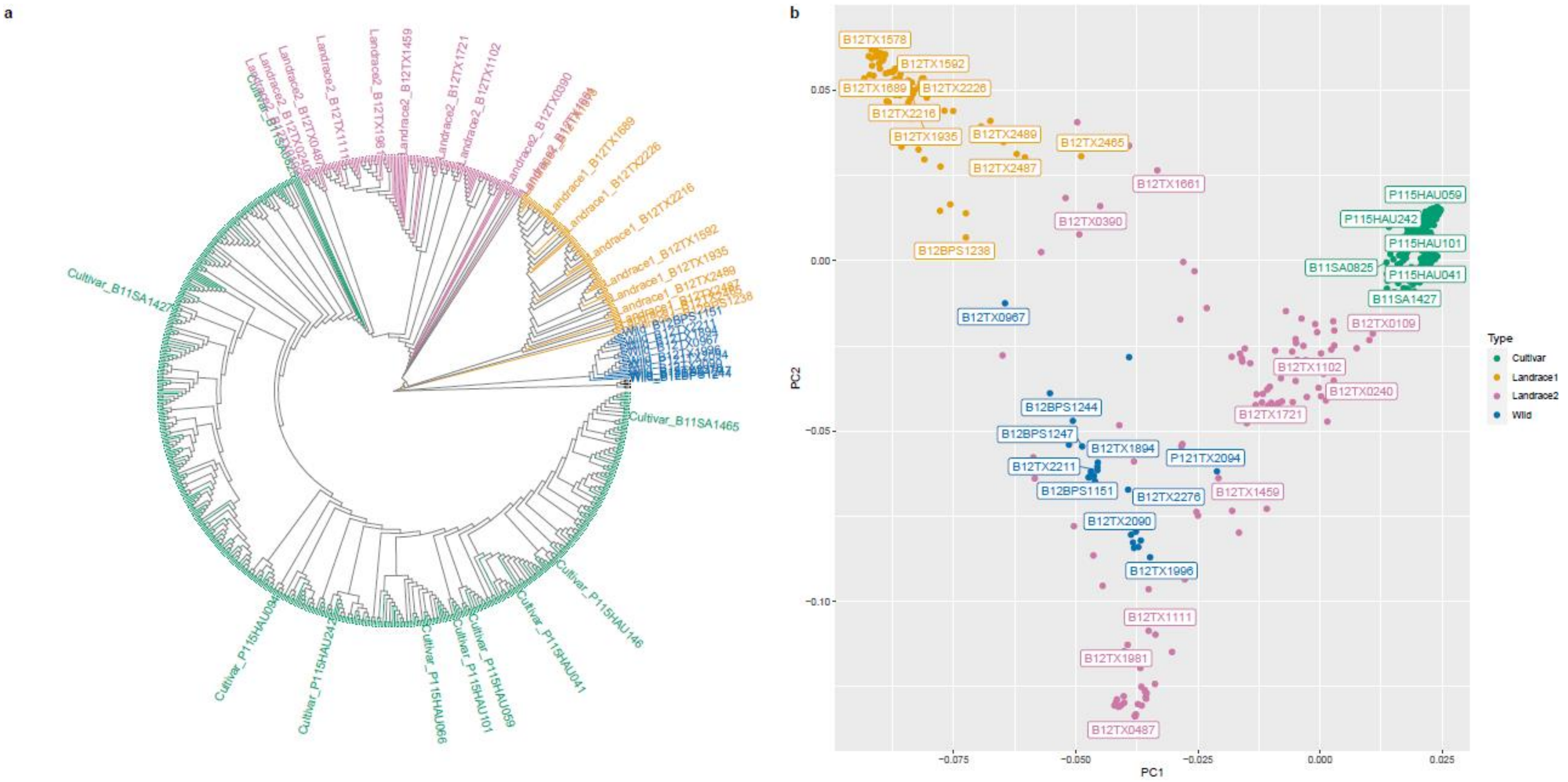

Figure S1. Phylogenetic tree a) and PCA plot b) adapted from Yuan et al. [15] (original files see: https://github.com/Wendellab/BYUReseq). The colored tree terminal branches and PCA dots represent different designated groups, wild (blue), landrace1 (yellow), landrace2 (pink) and cultivar (green). The samples selected for this study are labeled with individual ID.

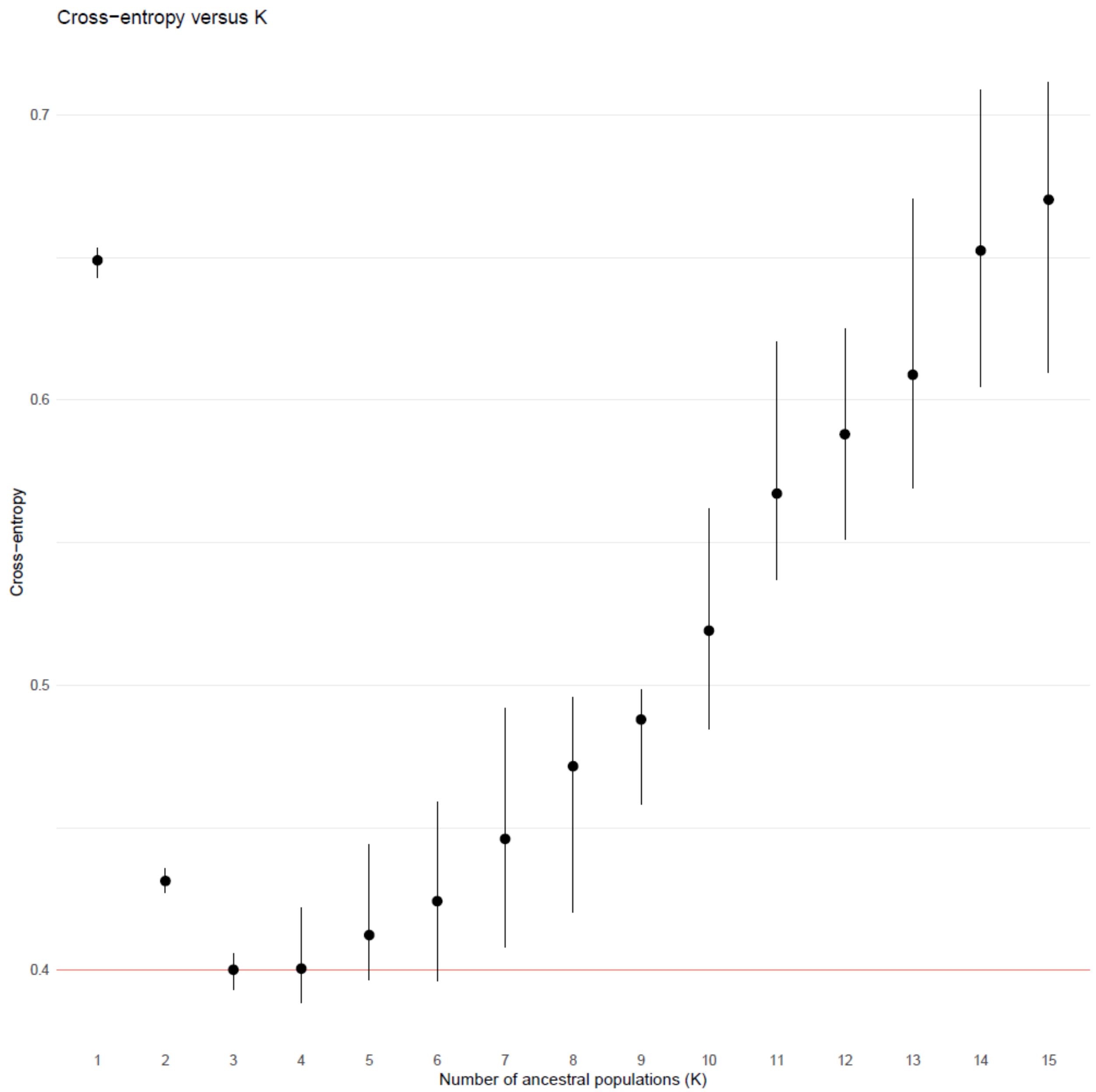

Figure S2. Cross-entropy criterion for LEA population structure determination. The average cross-entropy value for each K (the number of ancestral populations) represented by a dot with a vertical line that showed the standard divination between 10 replicates. The red horizontal line represents the lowest average cross-entropy value.

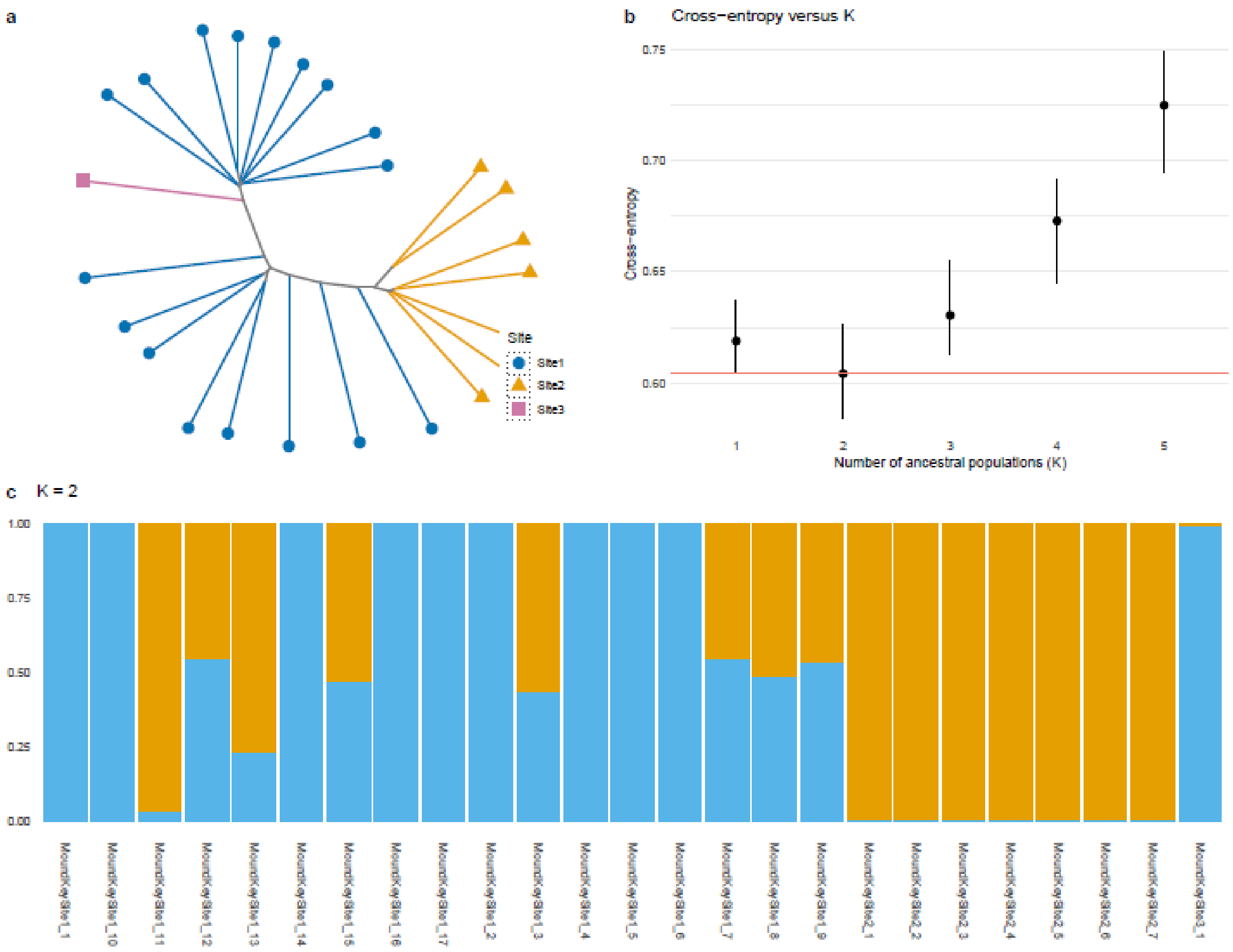

Figure S3. Population variations between 25 individuals collected over three sites from the Mound Key. a) Each site is distinguished by a different color and shape in PCA. b) Cross-entropy criterion for LEA ancestral population structure determination. c) LEA genetic structure for two ancestral populations (K = 2).
